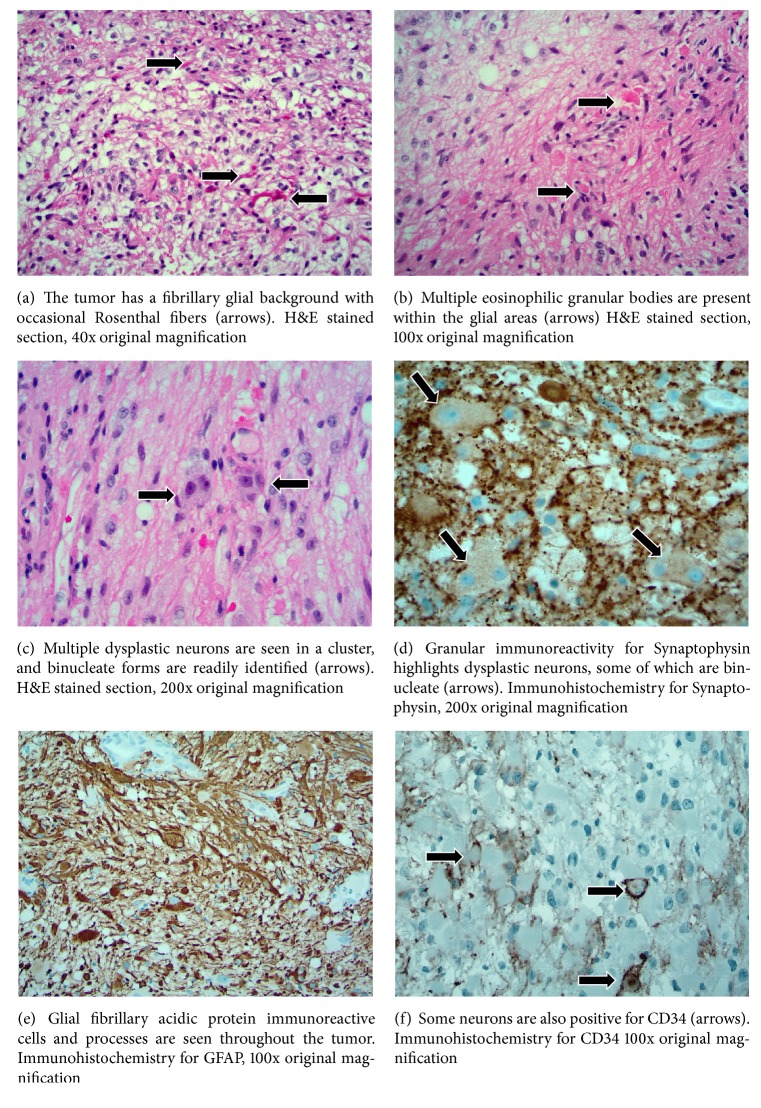# Suprasellar Ganglioglioma: Expanding the Differential Diagnosis

**DOI:** 10.1155/2018/9486064

**Published:** 2018-02-01

**Authors:** Isabella Tondi Resta, Arminder Singh, Bruce C. Gilbert, Mumtaz V. Rojiani, Cargill Alleyne, Amyn M. Rojiani

**Affiliations:** ^1^Department of Pathology, Augusta University-Medical College of Georgia, 1120 Fifteenth Street, Augusta, GA 30912-3600, USA; ^2^Department of Neurology, Augusta University-Medical College of Georgia, 1120 Fifteenth Street, Augusta, GA 30912-3600, USA; ^3^Department of Radiology, Augusta University-Medical College of Georgia, 1120 Fifteenth Street, Augusta, GA 30912-3600, USA; ^4^Department of Medicine, Augusta University-Medical College of Georgia, 1120 Fifteenth Street, Augusta, GA 30912-3600, USA; ^5^Department of Neurosurgery, Augusta University-Medical College of Georgia, 1120 Fifteenth Street, Augusta, GA 30912-3600, USA

## Abstract

This case study describes a young man with symptoms suggestive of the presence of a space-occupying lesion within the cranial cavity. Imaging studies confirmed a lesion in the suprasellar region and surgical intervention to remove the tumor yielded an unexpected diagnosis. Neuroimaging characteristics and histopathology including immunohistochemistry are described. Gangliogliomas are uncommon CNS neoplasms and are most commonly found in the temporal and frontal lobes of young, male adults. They are rarely seen in the suprasellar region and only a handful of cases have been reported to date. The differential diagnoses associated with these suprasellar region lesions can be dependent on the age of the patient and neuroimaging characteristics. The present report highlights the importance of histopathological examination and the need to consider a wide range of diagnostic entities in the differential diagnosis of lesions in this topographic distribution, including rarely encountered tumors such as gangliogliomas.

## 1. Clinical History

**Figure 1:**
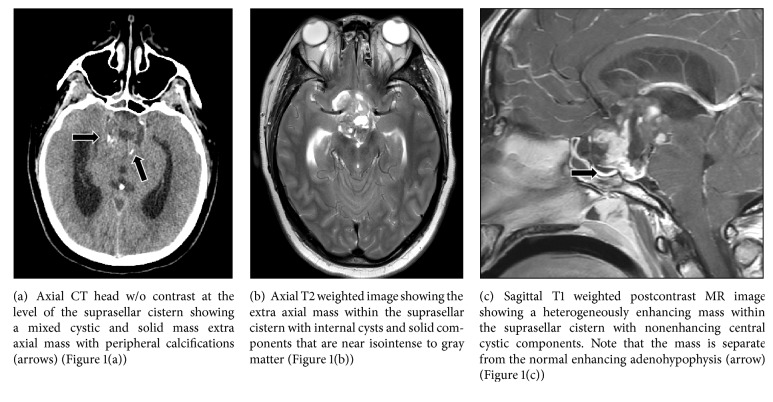


25-year-old Caucasian male with past medical history of headache presented to an outside hospital for headache and vomiting. He was confused and combative. CT head without contrast at an outside facility was reported as hydrocephalous with possible aqueduct stenosis. He was intubated for airway protection. He was transferred to this medical center for higher level of care. On arrival, he was sedated, pupils 2 mm sluggishly reactive to light, and oculocephalic, corneal, cough, and gag reflex were present. He withdrew each extremity on application of pain stimulus. Deep tendon reflexes were recorded as 2+. At admission axial CT of the head without contrast demonstrated an extra axial mass at the level of the suprasellar cistern, with mixed cystic and solid components and some peripheral calcification (black arrows) (Figure 1(a)).

An emergent external ventricular drain was placed for CSF diversion and the patient was admitted to Neuro-ICU. Axial T2 weighted MR image showed the extra axial mass within the suprasellar cistern with internal cysts and solid components that are near isointense to gray matter (Figure 1(b)). MR imaging studies with intravenous Gadolinium were performed. Sagittal T1 weighted postcontrast image showed a heterogeneously enhancing mass within the suprasellar cistern with nonenhancing central cystic components. This cystic and solid mass was separate from the normal enhancing adenohypophysis (arrow) (Figure 1(c)), with marked mass effect, superior and posterior displacement of the hypothalamus. The tumor extended into the prepontine and interpeduncular cisterns. The mass measured approximately 3.6 cm transversely and 4.2 cm in the anteroposterior plane. Given the location and imaging characteristics the primary diagnostic consideration was craniopharyngioma, although germ cell neoplasm, hypothalamic glioma, and meningioma were less likely candidates.

During the patient's ICU admission, endocrinology was consulted to evaluate pituitary function. Prolactin, cortisol, LH, FSH, and IGF-1 levels were normal, while testosterone levels were decreased (35 ng/dl). ACTH stimulation test was performed and reported as normal. On day 4 of hospitalization, he underwent a right pterional craniotomy with image guidance for resection of tumor. The tumor was only partially resected primarily due to its adherence to optic tracts and the internal carotid artery. Postoperatively he was treated for aspiration pneumonia and subsequently extubated on postoperative day 5. At this point his headache had improved significantly and neurological exam revealed a partial right 3rd cranial nerve palsy and bilateral papilledema. The extraventricular drain was removed on postoperative day 10 and the patient was discharged home on day 14.

## 2. Pathology/Biopsy Report

Microscopic sections revealed a tumor that had a predominantly glial background. There was a well-defined fibrillary glial proliferation, with occasional fragments of Rosenthal fibers similar to that seen in a pilocytic astrocytoma (Figure 2(a)). Multiple eosinophilic granular bodies were also present (Figure 2(b)). No mitotic activity was seen and the cells demonstrated only mild pleomorphism. Normal and dysplastic neurons were seen, interspersed within the fibrillary matrix. In some areas the neuronal population was present in clusters while in other areas these dysplastic cells were identified by their large size, occasionally ballooned appearance as well as the binucleate morphology of some cells (Figure 2(c)). Immunohistochemistry for GFAP readily identified the glial background with extensive staining of processes and cytoplasm (Figure 2(d)). Synaptophysin decorated the large occasionally binucleate cells, confirming their neural origin (Figure 2(e)). Some neurons were also immunoreactive for CD34 (Figure 2(f)). Collectively the morphologic features and immunophenotype confirmed the diagnosis of ganglioglioma, despite the lesion's unusual location.

## 3. Discussion

Gangliogliomas are defined in the WHO Classification of Tumors of the Central Nervous System as “a well-differentiated, slow growing, glioneuronal neoplasm composed of dysplastic ganglion cells (i.e. large cells with dysmorphic neuronal features without the architectural arrangement or cytological characteristics of cortical neurons) in combination with neoplastic glial cells” [1]. These tumors, which are composed of a combination of neuronal and glial tissue, have been reported to account for 0.4% of all CNS tumors and 1–1.5% of all brain tumors [2–4]. Though rare, they have been classified as grade I tumors because of their slow growing and mostly benign nature [5, 6]. Gangliogliomas in general are low grade neoplasms with a 7.5 year recurrence-free survival rate of 97% [4].

Gangliogliomas are most commonly found in the temporal and frontal lobes of young, male adults [7]. Their presence in the suprasellar region is rare, with only a handful of cases having been reported to date [8]. The differential diagnoses associated with these suprasellar region lesions can be dependent on the age of the patient. In adults, the differential diagnoses could include meningioma and pituitary adenoma with a suprasellar extension, while in children the list includes craniopharyngioma and hypothalamic chiasmatic glioma [9]. Other diagnoses that should be considered are tumors that occur more commonly in the suprasellar region, such as germinoma or eosinophilic granuloma [9]. The clinical presentation of a ganglioglioma will include seizures, and those that are present in the suprasellar area will additionally include visual impairment [9]. There have been two cases that reported diabetes insipidus caused by a ganglioglioma in the suprasellar region [8].

CT scan imaging of gangliogliomas has been reported to show a hypodense lesion with calcifications, and these lesions may enhance with contrast in fewer than 40% of patients [6]. Some cases of gangliogliomas have been reported to be undetectable by CT scans, and therefore MR imaging is strongly recommended for diagnosis [10]. MR imaging has shown that gangliogliomas may be made up of cystic or solid parts that appear differently on T1-weighted and T2-weighted images [6]. Approximately 57% of gangliogliomas have cystic components, which appear as hyperintense on T2-weighted images [9]. Cystic components of these tumors are distinguishable from more common cysts by their irregular margins and associated soft tissue components [6]. Solid tumor components appear isointense to hyperintense to gray matter on T2 weighted images but hypointense to fluid signal cystic components [9].

The histopathology of gangliogliomas is typically quite characteristic and is usually easily distinguishable from other diagnostic considerations. The unusual location of this tumor notwithstanding, the pathologic diagnosis was readily apparent. The presence of a diffuse neoplastic fibrillary glial background with eosinophilic granular bodies, combined with dysplastic ganglion cells, is quite characteristic. Binucleate neurons, often arranged in clusters with apparent disregard for normal configuration, are confirmatory. Additionally, immunohistochemistry for GFAP defined the glial component. Multiple dysplastic neurons immunoexpressed CD34: this marker is not expressed in normal neurons but is frequently seen in abnormal neurons in gangliogliomas [11].

The overall characteristics of gangliogliomas suggest that they are often well defined, which makes complete surgical excision a promising possibility for treatment with good prognosis [6, 10]. However, those that are found in the suprasellar region are often not easily accessible and subtotal resection is not uncommon, thus making radiotherapy the most preferred plan of treatment [9]. Because total removal of these suprasellar gangliogliomas can be difficult, the prognosis has been varied. Most patients who have been successfully treated have been stable since completion of treatment, with 80% having complete seizure relief and 20% experiencing a significant improvement of seizure symptoms [6]. However, there has been no record of improvement of vision in patients [9].

In summary this report presents a rare case of suprasellar ganglioglioma in a young male. The vast majority of gangliogliomas are found in the temporal and frontal lobes with only a few reports of occurrence in the suprasellar region. The differential diagnosis of lesions encountered in this region often does not include these tumors by either radiology or pathology. Increased awareness of the possibility of this rare lesion in a highly uncommon location is therefore important in the management of these tumors.

## Figures and Tables

**Figure 2:**